# Pharmacogenetic Testing in Treatment-resistant Panic Disorder: a Preliminary Analysis

**DOI:** 10.2174/0117450179337258241031035148

**Published:** 2024-12-03

**Authors:** Marcos Fidry, Morena Mourao Zugliani, Mariana Costa do Cabo, Renan Machado Martins, Manuella Assad Gomez, Clara Gitahy Falcão Faria, Antonio Egidio Nardi, Rafael C. Freire

**Affiliations:** 1 Laboratory of Panic and Respiration, Instituto de Psiquiatria, Universidade Federal do Rio de Janeiro (UFRJ). Rio de Janeiro, RJ, Brazil; 2 Department of Psychiatry and Centre for Neuroscience Studies, Queen’s University. Kingston, ON, Canada; 3 Kingston General Hospital Research Institute, Kingston Health Sciences Centre. Kingston, ON, Canada

**Keywords:** Treatment-resistant anxiety disorder, Folic acid, Pharmacokinetics, Neuropharmacology, Cytochrome P450, Methylenetetrahydrofolate reductase, ABCB1 protein

## Abstract

**Background:**

Many pharmacological treatments are considered effective in the treatment of panic disorder (PD), however, about 20 to 40% of the patients have treatment-resistant PD. Pharmacogenetics could explain why some patients are treatment-resistant.

**Objective:**

Our objective was to gather preliminary data on the clinical usefulness of pharmacogenetic testing in this disorder.

**Methods:**

Twenty patients with treatment-resistant PD were included in this observational study and submitted to commercial pharmacogenetic testing. Testing panel included gene polymorphisms related to CYP, genes *EPHX1*, *UGT1A4*, *UGT2B15*, *ABCB1*, *ADRA2A*, *ANKK1*, *COMT*, *DRD2*, *FKBP5*, *GRIK4*, *GSK3B*, *HTR1A*, *HTR2A*, *HTR2C*, *MC4R*, *OPRM1*, *SCN1A*, *SLC6A4* and *MTHFR*. Participants received treatment-as-usual for PD before being enrolled in this study, including first-line and second-line medications for PD.

**Results:**

In 30% of the patients, the tests indicated reduced chance of response to the prescribed drug, while they indicated very low serum levels of the prescribed drug in 20% of the subjects. The pharmacogenetic tests predicted reduction of MTHFR enzyme activity in 74% of the patients. ABCB1 gene alleles associated to drug resistance were found in 90% of the samples.

**Conclusion:**

Commercial pharmacogenetic testing failed to predict negative treatment outcome in most patients with PD. The association between treatment-resistance in PD and the genes CYP2C19, MTHFR and ABCB1 deserves further study.

## 1 INTRODUCTION

Guidelines recommend multiple treatment options for panic disorder (PD), including antidepressants (AD) and benzodiazepines (BZD). These recommendations are based mainly on meta-analysis and randomized controlled clinical trials [1-3]. Although treatment options are effective for most patients, many of them fail to respond to treatment and achieve remission. Clinical trials with selective serotonin reuptake inhibitors (SSRI) with a duration between 8 and 12 weeks demonstrated nonresponse to treatment in 17-61% of patients [4, 5]. Another concern related to pharmacological treatments is the risk of adverse events and side effects. In 2018, a meta-analysis found the risk ratio of dropping out due to adverse events comparing AD and placebo was 1.49 (CI: 1.25-1.78) [6]. Approximately 40% to 20% of patients with PD are treatment-resistant [7-10], adequate treatments for these patients are an unmet need. A better understanding of how genes, PD, and treatments interact would make personalized treatments possible, increasing treatment success rates and decreasing the prevalence of treatment-resistant PD.

There is evidence of the role of genetics on PD pathogenesis and clinical manifestations [11, 12]. A meta-analysis of family and twin studies indicates that PD is highly familial, and suggests a heritability of about 48% [13]. The valine allele of the Val158Met catechol-O-methyltransferase (*COMT*) polymorphism or a nearby locus was associated with the physiopathology of PD [14]. A study using a family-based association test [15] generated nominal support for allelic association in the tachykinin receptor 1 gene (*TACR1*) and gastrin-releasing peptide gene (*GRP*) with PD. The study of genes related to pharmacological treatments is a promising field of research, and pharmacogenetics will likely become in the future a useful tool in personalized psychiatry. Currently, there are very few studies on anxiety disorder or anxiolytic medications [16-18]. Polymorphisms in the Methylene- tetrahydrofolate reductase (*MTHFR*) genes, Cytochrome P450 (CYP) genes and ATP-binding cassette sub-family B member 1 (*ABCB1*) genes are promising pharmacogenetic targets.

MTHFR is a key enzyme for the critical process of one-carbon metabolism involving folate and homocysteine metabolisms [19]. MTHFR single nucleotide poly- morphisms (SNP) with lower enzyme activity phenotypes has been associated with many psychiatric disorders, including major depressive disorder and bipolar disorder [20]. These SNPs may also be associated with anxiety disorders, although literature about this topic is scant [21, 22]. The literature also indicates that folate deficiency is associated to poor response to treatment in patients with major depressive disorder (MDD) [23]. In addition, clinical trials with treatment-resistant MDD showed that the administration of 15 mg of L-methylfolate – which bypasses the MTHFR in the folate pathway – as an add-on to antidepressants is effective for these patients [24]. The authors speculate that, since MTHFR deficiency is associated with poor response to treatment in MDD patients, it could also be associated with treatment-resistant PD. In this case, testing for *MTHFR* polymorphisms could be useful in the identification of PD patients with MTHFR deficiency and in adjusting the treatment accordingly.

CYP superfamily of enzymes responsible for the pharmacokinetics of most of AD and anxiolytics, include the enzymes CYP3A4, CYP3A5, CYP2C19, and CYP2D6 [25-27]. There are multiple SNPs for these enzymes; and with phenotypical variation of enzyme activity, there are differences in drug metabolism, consequentially [28]. Intermediate and poor metabolism means decreased drug metabolism, which was associated with more adverse events and side effects, while rapid and ultrarapid metabolism means increased drug metabolism, which was associated with decreased effectiveness [29]. Some patients with PD may eventually be labeled as treatment-resistant due to abnormal activity levels of CYP enzymes. A given pharmacological treatment could have no effect or very small effect on a patient who is a rapid or ultra-rapid metabolizer. On the other hand, if a patient is a poor metabolizer of a given drug, there is a high risk of intolerable side effects.

For an antidepressant to exert its effects it must reach the central nervous system, and the P-glycoprotein has an important role in transporting these medications across the blood-brain barrier. This glycoprotein, which is also known as multidrug resistance protein 1 and ABCB1, is encoded by the *ABCB1* gene. Since SNPs in the *ABCB1* gene have been associated to poor response to treatment with antidepressants in MDD [30], the authors extrapolate that this gene could also be associated to poor response to antidepressants in patients with PD.

Commercial pharmacogenetic testing is used to identify SNPs associated with multiple enzymes which are important for drug metabolism, such as the CYP superfamily. The testing panels also include SNPs for other enzymes such as MTHFR and COMT and other proteins such as P-glycoprotein, serotonin receptors and transporters. Pharmacogenetic testing may save treatment costs and increase treatment adherence [29]. There are few studies on pharmacogenetics with PD patients, and little is known about the effects of SNPs in clinical presentations. There is not enough information on the effects of SNPs in response to treatment [16, 18], but certain SNPs may be associated with poor response to treatment in patients with PD. Pharmacogenetic testing in treatment-resistant PD may aid the clinician by elucidating which genetic factors are contributing to treatment resistance in their patients, and they may tailor the treatment accordingly [18].

Our primary objective was to ascertain if the pharmacogenetic test panel would predict the negative outcome of pharmacological treatments administered to patients with PD. The secondary objective was to identify which abnormal SNPs would eventually explain non-response to pharmacological treatment in these patients.

## 2 MATERIALS AND METHODS

This was a retrospective study with 20 patients between 18 and 60 years old, from both sexes, who had received treatment-as-usual (TAU) for PD. These patients were invited to participate in the current study only after completing 8 weeks of standard-of-care pharmacological treatment for PD.

All participants were recruited from a subsample of treatment non-responders from another study (unpublished data). Sociodemographic and clinical data, including scores from clinical scales, were retrieved from the study file. In the previous study, all patients were evaluated with the MINI v.4.417 [31]. Diagnoses were confirmed in a clinical interview with an experienced psychiatrist (MF or RCF), based on DSM-IV criteria for PD. Inclusion criteria were: age from 18 to 60 years, both sexes, and confirmed diagnosis of PD. Exclusion criteria were: patients taking psychiatric medications at baseline, patients on cognitive behavioral therapy at baseline, illiterate patients, patients with psychotic symptoms, severe personality disorders, intellectual impairment, or severe clinical or neurological diseases. Patients were not allowed to receive cognitive behavioral therapy during the 8 weeks of TAU. The clinical scales administered were: Panic and Agoraphobia Symptoms (PAS) [32], Beck Anxiety Inventory (BAI) [33], Beck Depression Inventory (BDI) [34], Clinical Global Impression Severity (CGI-S) and Improvement (CGI-I) [35]. CGI-I scores from 1 to 7 mean “very much improved”, “much improved”, “minimally improved”, “no change”, “minimally worse”, “much worse” and “very much worse”, respectively. Participants were initially recruited from the Anxiety Disorder Clinic in the Institute of Psychiatry of the Federal University of Rio de Janeiro. This was a convenience sample.

The authors reviewed the database from the previous study. Patients with CGI-I higher than 2 (which corresponds to “minimally improved” or worse outcome) after 8 weeks of treatment were considered treatment-resistant patients. From the list of 41 treatment-resistant patients, contact information was outdated for 5 of them, and they could not be reached. Thirty-six people were invited to participate in the current study, but 16 did not agree to participate. Patients who consented to participate in the current study agreed that their data from the previous study was retrieved.

All participants were submitted to pharmacogenetic analysis with a standard commercial pharmacogenetics testing kit by GnTech© (http://www.gntech.med.br). Material was collected and sent to the laboratory according to their guidelines. As a summary of the results report, there was one of three possible recommendations for each drug: “use according to label” – meaning there were no concerns regarding the given medication; “use with attention” – meaning there were minor concerns regarding the given medication, with a slight risk of SNPs interfering with the medication concentration; and “use with caution and attention” – meaning there were concerns regarding the given medication, with risk of SNPs interfering with the medication concentration, causing adverse events or being not effective. The key enzymes’ genetic polymorphisms evaluated were: *CYP2D6, CYP2C19, CYP2C9, CYP1A2, CYP3A4, CYP3A5, CYP2B6, FKBP5, HTR2A, ANKK1, HTR1A, HTR2C, DRD2, GRIK4, ADRA2A, OPRM1, COMT, SLC6A4 e ABCB1, FKBS, GSK3B, EPHX1, UGT1A4, UGT2B15, MC4R, SCN1A, SLC6A4, MTHFR* (rs1801131 and rs1801133). All genes listed above are the ones included in the standard commercially available kit from GnTech©, which reflects real-world use of pharmacogenetics testing. All analyses had at least level 2B of evidence according to PharmGKB [36]. Level 2B clinical annotations must be supported by at least two independent publications, but they describe variant-drug combinations with a moderate level of evidence supporting the association. Some of these SNPs are classified as tier 1, Very Important Pharmacogenes (VIP) in PharmGKB and included in the guidelines from the Clinical Pharmacogenetics Implementation Consortium (CPIC) [37].

SNPs in CYP-related genes, *EPHX1*, *UGT1A4, and UGT2B15,* predict the bioavailability of the enzymes, consequentially predicting the metabolism speed of drugs metabolized by these enzymes. According to each gene, the drug metabolism prediction could range from ultrarapid metabolism to poor metabolism. SNPs in genes *ANKK1, HTR2C, MC4R,* and *OPRM1* predict the risk of side effects and adverse events in the presence of certain drugs. SNPs in genes *ABCB1, ADRA2A, COMT, DRD2, FKBP5, GRIK4, GSK3B, HTR1A, HTR2A, SCN1A* and *SLC6A4* predict response to treatment with certain medications. SNPs in the *MTHFR* gene predict the activity of the MTHFR enzyme.

Due to the study design, only descriptive statistical analysis was conducted. There was no sample size calculation since this was a pilot study.

This study was approved by the local ethics committee, the Research Ethics Board of the Institute of Psychiatry of the Federal University of Rio de Janeiro (Comite de Etica em Pesquisa do Instituto de Psiquiatria da Universidade Federal do Rio de Janeiro) prior to initiation of research work. The study was performed according to local guidelines and regulations. All participants received a letter of information and provided written informed consent before being included in the study. A code was assigned to each participant. Information collected for the study participants was stored as hardcopy in locked cabinets and as coded electronic copies in secure servers in the Anxiety Disorder Clinic. The biological samples were sent to the laboratory with the code for each participant, no additional information about the participant was attached. Results came back from the laboratory as hardcopy and transcribed by the research team to the secure electronic files in the Anxiety Disorder Clinic.

## 3 RESULTS

Although all patients were non-responders, there was a general decrease of 32% on the PAS score, 21% on the CGI-S score, 18% on the BAI score, and 15% on the BDI score. Only 1 patient had worse scores after the 8 weeks of treatment. All the patients were on AD, and 60% (12 patients) were using adjuvant BZD. Forty percent (n=8) were on SSRI, 40% (n=8) were on TCA and 20% (n=4) were on venlafaxine. Patients were on one of the following AD: clomipramine, escitalopram, fluoxetine, imipramine, paroxetine, sertraline, or venlafaxine. The dose for each antidepressant was calculated in equivalents of fluoxetine (Table **1**) [38, 39]. Two patients were taking a dose lower than the equivalent to 20 mg of fluoxetine due to tolerability issues. BZD used were alprazolam, bromazepam, and clonazepam. The main sociodemo- graphic and clinical characteristics are displayed in Table **1**.

Confronting the pharmacological treatment each patient received and the recommendations from the pharma- cogenetic tests, the general recommendations from the pharmacogenetic report were “use according to the label” in 40% (n=8), “use with attention” in 55% (n=11), and “use with caution and attention” in only one case (5%). The test predicted a high serum level of AD in use in 30% of the patients (n=6), a low serum level in 20% (n=4), and the expected serum level in 50% of the patients. In addition, the pharmacogenetic test reports predicted reduced response to the pharmacological treatment in 30% (n=6) of the patients and a high risk of side effects in none of the patients, for the given AD. Regarding the two patients taking low doses of AD, the report did not predict high serum levels of the drug or increased risk of side effects.

Enzymatic activity phenotypes associated with SNPs are shown in Table **2**, other SNPs possibly affecting response to treatment are shown in Table **3**. Considering the two genes for MTHFR, the pharmacogenetic testing report predicted some degree of enzyme deficiency in 74% (n=14) of the patients, being a more severe form of MTHFR deficiency present in 16% (n=3) of the patients. There was an overlap between the two *MTHFR* genes (rs1801131 and rs1801133) with heterozygosis in 16% (n=3) of the patients, but no overlap for homozygosis for the variant alleles. Another relevant result was the gene for permeability glycoprotein ABCB1 predicting poor response to treatment in 90% (n=18) of the patients.

## 4 DISCUSSION

Pharmacogenetic testing could not predict the negative outcome in most cases. Regarding pharma- cokinetics, the serum level predicted by pharmacogenetic testing was normal in 50% of patients. The CYP3A4 and CYP2D6 activity was predicted normal for most patients. CYP2C19 phenotype predicted slower metabolism in 25% and faster metabolism in 40% of the patients, with only 35% of patients showing normal enzyme activity. Studies conducted in the Brazilian population [40] showed that ethnicity plays a significant role in the prevalence of *CYP2C19* polymorphisms. Since clomipramine, imipra- mine, escitalopram, fluoxetine, and sertraline are substrates of CYP2C19, altered metabolism could have contributed to tolerability or effectiveness issues in some patients.

Although not expected, most patients had *MTHFR* polymorphisms, with a predicted reduction of MTHFR enzyme activity. Most of them showed mild impairment, and less than half of the sample showed more prominent impairment in enzyme activity. This finding cannot be explained by Brazilian genetic distribution, since rs1801131 allele G is found in 29% of the general white population and 17% of the African descendant population (*versus* 41.7% and 62.5% in the current study), and rs1801133 allele A is found in 31% of the general white population and in 23% of African descendant population (*versus* 41.7% and 25% in the current study) [41]. This may indicate a role of MTHFR enzymatic activity in PD treatment response and even on PD symptoms. Genetic polymorphisms of *MTHFR* – which catalyzes the conversion of methylenetetrahydrofolate to the active form of the vitamin 5-methyltetrahydrofolate – interfere in the conversion of folate to the active form of this vitamin, being folate deficiency the result. The association between folate deficiency and MDD is well established, including poor response to treatment in patients with MDD and folate deficiency. Low MTHFR activity is likely associated with treatment resistance in MDD as well [42, 43]. However, the association of folate metabolism with anxiety disorders is still unclear. Given the significant overlap between neurobiological findings in mood disorders and anxiety disorders, one would suspect folate metabolism to have a relevant role in anxiety disorders too, including PD. In addition, a recent meta-analysis [44] showed that supplementation with folate was effective as an adjunctive treatment for major depressive disorder, but there are no studies demonstrating the effectiveness of this treatment in anxiety disorders at present. Future studies should determine the role of MTHFR in PD, these studies should include patients with non-treatment-resistant PD and healthy subjects.

In the studied sample, 90% of participants had the allele G in the *ABCB1* (rs2032582) gene, which is higher than expected for the Brazilian population. Estrela, Ribeiro [45], found allele G in 61% of white, 70% of intermediate (brown), and 81% of black Brazilians. Genetic variation at the *ABCB1* locus has been studied as a predictor of treatment outcomes for several medications [30]. This gene encodes the P-glycoprotein, which is involved in the active transport of several commonly prescribed antidepressants through the blood-brain barrier. Polymorphisms in this gene could interfere with the concentration or function of P-glycoprotein, and this would alter brain concentrations of substrate medications, which include many antidepressants. In a clinical trial with MDD, Schatzberg, and DeBattista [30] found that one SNP from the *ABCB1* gene had a significant effect on remission rates and side effects frequency in patients treated with escitalopram, sertraline or venlafaxine.

The main limitation of the study was the lack of control groups (healthy participants and non-treatment-resistant PD patients). Due to the exploratory nature of the current study, the sample size was small. Another limitation was the current study was geared toward measuring pharmacological treatment effectiveness, while there were no systematic assessments of side effects and other adverse events. There were no dropouts due to tolerability issues, though. It was not possible to confront the actual side effects and adverse events to the predictions from pharmacogenetic tests. The sample included in this study is not representative of all patients with treatment-resistant PD because it was a clinical convenience sample, in which most patients received multiple treatments previously and presented with severe PD initially. Another limitation of the current study was the low level of evidence for many of the genes included in the panel. Only the genes for *CYP 2D6, 2C19, 2C9, 3A4, 3A5, 2B6, MTHFR, ABCB1, COMT* and *DRD2* have high level of evidence in the literature [46]. Future studies should compare the pharmacogenetic features of treatment-resistant and treatment-responsive PD patients, and these studies should have adequate sample size and power to detect differences between these two groups. Since genetic features seem to be associated with treatment resistance across different psychiatric disorders, future studies should also include participants with other anxiety disorders and mood disorders.

## CONCLUSION

Commercial pharmacogenetic testing could not predict the negative outcome of pharmacological treatment in most of the patients with PD in the current study. The SNPs in *CYP2C19, MTHFR,* and *ABCB1* genes may have interfered to some extent with treatment tolerability or effectiveness. These three genes deserve further investigation as possible contributing factors to treatment resistance in PD. If large-scale studies confirm the relevance of SNPs in *CYP2C19, MTHFR,* and *ABCB1*, this would be an important step in the advancement of personalized psychiatry. In the future, pharmacogenetic testing may become an important tool for clinicians, making it possible to tailor treatments according to the patient’s features.

## Figures and Tables

**Table 1:** Clinical and sociodemographic characteristics of the sample.

-	**Mean/ N**	**SD/ %**	**Minimum**	**Maximum**
Sex	-	-	-	-
Male	5	25%	-	-
Female	15	75%	-	-
Age (in years)	38.2	7.88	24	54
Marital Status	-	-	-	-
Married	10	50%	-	-
Non-Married	10	50%	-	-
Employment	-	-	-	-
Employed	11	55%	-	-
Unemployed	9	45%	-	-
Years of education	12.85	3.83	5	18
Income (in minimum wages)	3.73	1.69	1	7
Ethnicity (self-declaration)	-	-	-	-
White	12	60%	-	-
African descendant	8	40%	-	-
Previous treatments	1	1.12	0	4
0	8	40%	-	-
1	7	35%	-	-
More than 1	5	25%	-	-
Pharmacological treatment	-	-	-	-
Clomipramine	2	10%	-	-
Imipramine	6	30%	-	-
Escitalopram	1	5%	-	-
Sertraline	1	5%	-	-
Fluoxetine	4	20%	-	-
Paroxetine	2	10%	-	-
Venlafaxine	4	20%	-	-
Dose in fluoxetine equivalents (mg)	29	13	7	60
**Baseline visit**	-	-	-	-
CGI-S	5.05	0.85	3	7
BAI	40,7	9.17	19	59
BDI	25.55	10.23	9	41
PAS	29.65	7.79	9	39
**8-week visit**	-	-	-	-
CGI-S	4	1.02	3	6
CGI-I	3.05	0.94	2	6
BAI	33.25	14.94	4	52
BDI	21.75	9.37	9	38
PAS	20.15	10.50	2	39

**Table 2:** Enzymatic activity phenotypes associated to single nucleotide polymorphisms.

-	**N** **(N = 20)**	**Percentage of Valid Cases**	**Genes Responsible for Altered Metabolism**
CYP2D6 *	-	-	-
Normal	19	95%	-
Ultrarapid	1	5%	Duplicated 2D6 gene
CYP2C19 *	-	-	-
Poor	1	5%	Heterozygous with two variant alleles (2A/35)
Intermediate	4	20%	Heterozygous for variant allele (2A/-)
Normal	7	35%	-
Rapid	7	35%	Heterozygous for variant allele (17/-)
Ultrarapid	1	5%	Homozygous for variant allele (17/17)
CYP2C9 *	-	-	-
Intermediate	3	15%	Heterozygous for variant alleles (2/- or 3/-)
Normal	17	85%	-
*CYP1A2*	-	-	-
Normal	20	100%	-
CYP3A4 *	-	-	-
Normal	20	100%	-
CYP3A5 *	-	-	-
Poor	10	50%	Homozygous for one variant allele (3A/3A) or heterozygous for two variant alleles (3A/3E)
Intermediate	10	50%	Heterozygous for variant alleles (6/-, 3A/- or 3E/-)
Normal	0	0%	-
CYP2B6 *	-	-	-
Poor	1	5%	Homozygous for variant allele (9/9)
Intermediate	6	30%	Heterozygous for variant allele (9/-)
Normal	13	65%	-
*EPHX1*_rs1051740	-	-	-
Normal	9	45%	-
Rapid	11	55%	Heterozygous for variant allele (C/-)
*EPHX1*_rs2234922	-	-	-
Normal	14	70%	-
Rapid	6	30%	Heterozygous for variant allele (G/-)
*UGT1A4*	-	-	-
Intermediate	4	20%	Heterozygous for variant allele (2/- or 3A/-)
Normal	14	70%	-
Ultrarapid	2	10%	Heterozygous for variant allele (3B/-)
*UGT2B15*	-	-	-
Poor	2	10%	Homozygous for variant allele (5/5)
Intermediate	8	40%	Heterozygous for variant allele (5/-)
Normal	10	50%	-

**Note: **Ultrarapid = ultrarapid metabolizer genotype, genotype associated with very increased metabolism of drugs associated with the given enzyme; Rapid = rapid metabolizer genotype, genotype associated with increased metabolism of drugs associated with the given enzyme; Normal = normal metabolizer genotype, genotype associated to normal metabolism of drugs associated to the given enzyme; Intermediate = intermediate metabolizer genotype, genotype associated to decreased metabolism of drugs associated to the given enzyme; Poor = poor metabolizer genotype, genotype associated to very decreased metabolism of drugs associated to the given enzyme. * Tier 1, Very Important Pharmacogenes (VIP) in PharmGKB and included in the guidelines from the Clinical Pharmacogenetics Implementation Consortium (CPIC).

**Table 3:** Single nucleotide polymorphisms possibly affecting response to treatment.

-	**N (N = 20)**	**Percentage of Valid Cases**	**Genes Responsible**
*MTHFR*_rs1801131 (*MTHFR1298*) *	-	-	-
Missing	1	-	-
Poor	2	11%	Homozygous for variant allele (CC)
Intermediate	8	42%	Heterozygous for variant allele (AC)
Normal	9	47%	-
*MTHFR*_rs1801133 (*MTHFR677*) *	-	-	-
Missing	1	-	-
Poor	1	5%	Homozygous for variant allele (TT)
Intermediate	6	32%	Heterozygous for variant allele (CT)
Normal	12	63%	-
ABCB1 *	-	-	-
Favorable	2	10%	Homozygous for allele A (AA)
Unfavorable	18	90%	Homozygous for allele G (GG) or heterozygous (GA)
*ADRA2A*	-	-	-
Favorable	15	75%	Homozygous for allele G (GG) or heterozygous (GC)
Unfavorable	5	25%	Homozygous for allele C (CC)
*ANKK1*	-	-	-
High risk of SE	10	50%	Homozygous for allele A (AA) or heterozygous (GA)
Low risk of SE	10	50%	Homozygous for allele G (GG)
*COMT* rs13306278	-	-	-
Favorable	16	80%	Homozygous for allele C (CC)
Unfavorable	4	20%	Heterozygous (CT)
*COMT* rs4680 *	-	-	-
Favorable	17	85%	Homozygous for allele G (GG) or heterozygous (AG)
Unfavorable	3	15%	Homozygous for allele A (AA)
DRD2 *	-	-	-
Favorable	20	100%	Homozygous for allele T (TT) or heterozygous (TC)
Unfavorable	0	0%	Homozygous for allele C (CC)
*FKBP5*	-	-	-
Favorable	7	35%	Homozygous for allele A (AA) or heterozygous (AG)
Unfavorable	13	65%	Homozygous for allele G (GG)
*GRIK4*	-	-	-
Favorable	6	30%	Homozygous for allele C (CC)
Unfavorable	14	70%	Homozygous for allele T (TT) or heterozygous (TC)
*GSK3B* rs334558	-	-	-
Favorable	15	75%	Homozygous for allele G (GG) or heterozygous (AG)
Unfavorable	5	25%	Homozygous for allele A (AA)
*GSK3B* rs6438552	-	-	-
Favorable	4	20%	Homozygous for allele G (GG)
Unfavorable	16	80%	Homozygous for allele A (AA) or heterozygous (AG)
*HTR1A*	-	-	-
Favorable to milnacipran	16	80%	Homozygous for allele C (CC) or heterozygous (CG)
Favorable to paroxetine	4	20%	Homozygous for allele G (GG)
*HTR2A*	-	-	-
Favorable	9	45%	Homozygous for allele A (AA) or heterozygous (AG)
Unfavorable	11	55%	Homozygous for allele G (GG)
*HTR2C* rs1414334	-	-	-
High risk of SE	6	30%	Homozygous for allele C (CC) or heterozygous (CG)
Low risk of SE	14	70%	Homozygous for allele G (GG)
*HTR2C* rs3813929	-	-	-
High risk of SE	20	100%	Homozygous for allele C (CC) or heterozygous (CT)
Low risk of SE	0	0%	Homozygous for allele T (TT)
*MC4R*	-	-	-
High risk of SE	0	0%	Homozygous for allele A (AA)
Low risk of SE	20	100%	Homozygous for allele C (CC) or heterozygous (CA)
*OPRM1*	-	-	-
High risk of SE	1	5%	Homozygous for allele G (GG) or heterozygous (GA)
Low risk of SE	19	95%	Homozygous for allele A (AA)
*SCN1A*	-	-	-
Favorable	15	75%	Homozygous for allele C (CC) or heterozygous (CT)
Unfavorable	5	25%	Homozygous for allele T (TT)
*SLC6A4*	-	-	-
Favorable	18	90%	Homozygous for allele L (LL) or heterozygous (LXL or LC)
Unfavorable	2	10%	Homozygous for allele C (CC)

**Note: **SE= side effects. * Tier 1, Very Important Pharmacogenes (VIP) in PharmGKB and included in the guidelines from the Clinical Pharmacogenetics Implementation Consortium (CPIC).

## Data Availability

The data supporting the findings of the article is available in the Zenodo Repository at https://clinical-practice-and-epidemiology-in-mental-health.com/.

## LIST OF ABBREVIATIONS

AD: = Antidepressants
PD: = Panic Disorder
BZD: = Benzodiazepines
MTHFR: = Methylenetetrahydrofolate Reductase
SNP: = Single Nucleotide Polymorphisms
MDD: = Major Depressive Disorder

## STANDARDS OF REPORTING

STROBE guidelines were followed.
